# Prospective Evaluation of Symptom Burden and Medication Use in Seasonal Allergic Rhinitis/Rhinoconjunctivitis Patients Considering Allergen-Specific Immunotherapy

**DOI:** 10.3390/jcm15114035

**Published:** 2026-05-22

**Authors:** Anna Rybachuk, Christian Neuhof, Edmund Curtius, Cengizhan Acikel, Susann Fragel, Hacer Sahin, Nadine Katzke, Kijawash Shah-Hosseini, Silke Allekotte, Esther Raskopf

**Affiliations:** 1 ClinCompetence Cologne GmbH, 50668 Cologne, Germany; 2 Institute of Medical Statistics and Computational Biology, Faculty of Medicine, University of Cologne, Kerpener Str. 62, 50937 Cologne, Germany

**Keywords:** epidemiologic study, allergen immunotherapy, grass pollen allergy, seasonal allergic rhinitis/rhinoconjunctivitis, combined symptom–medication score

## Abstract

**Background/Objectives:** Allergen immunotherapy (AIT) is the only disease-modifying treatment for grass pollen allergy. However, the proportion of patients interested in AIT who meet guideline-defined eligibility criteria remains unclear. This study aimed to characterise symptom burden, medication use, and AIT eligibility in adult patients with grass pollen allergy during the peak pollen season. **Methods:** In this multicentre, prospective, non-interventional epidemiological study, 479 adults with confirmed grass pollen allergy recorded daily nasal, ocular, and systemic symptoms, as well as anti-allergic medication use, via a validated electronic diary (CCC STUDY Diary) over a 30-day period in June/July 2025. A combined symptom–medication score (CSMS) was calculated daily, with a predefined threshold of ≥1.5 indicating clinically relevant symptom severity and potential eligibility for AIT. Both additive and weighted calculation approaches for the CSMS and the daily medication score (dMS) were evaluated to assess methodological robustness and reproducibility. **Results:** The mean additive CSMS was 2.14, indicating moderate symptom burden. Overall, 63.3% of participants exceeded the CSMS threshold of 1.5 and were considered eligible for AIT. Sensitivity analyses demonstrated excellent concordance between additive and weighted CSMS/dMS calculations (Spearman’s ρ >0.98; *p* < 0.001), and Bland–Altman analysis confirmed minimal bias (0.157) and narrow limits of agreement. Asthma was reported as a comorbidity in 36% of patients, generally associated with mild to moderate daily respiratory symptoms. Limitations included the self-reported nature of the data and a slightly reduced sample size; however, the results are representative of adult patients seeking care in specialised allergy centres in Germany. **Conclusions:** The CSMS also in its additive and therefore modernised form is a reliable, reproducible, and clinically meaningful tool for quantifying symptom severity and identifying patients suitable for AIT. Approximately two-thirds of adults interested in grass pollen AIT exhibited moderate to severe symptoms and were eligible for treatment according to current guideline recommendations.

## 1. Introduction

Numerous clinical trials of allergen immunotherapy (AIT) targeting birch, grass, and house dust mite allergies have been unsuccessful. Notably, it appears that in over two-thirds of cases, patients expressing interest in AIT do not exhibit the moderate-to-severe symptoms recommended by current guidelines [1]. This finding stems from a large-scale clinical trial of subcutaneous AIT for house dust mite allergy, published in one of the world’s leading journals.

In 2014, the European Academy of Allergy and Clinical Immunology (EAACI) established a primary outcome parameter for clinical trials in AIT, replacing the previously mandated co-primary endpoints [2]. This symptom and medication assessment, further referred to as the “weighted” form of the combined symptom–medication score (CSMS), captures both nasal and ocular symptoms, as well as the symptomatic therapies required. This parameter has been in use for approximately a decade. With the aid of electronic diaries, most commonly implemented as mobile applications, patients record these parameters on a daily basis.

The primary objective of the present epidemiologic study was to determine the distribution of the mean CSMS in a cohort of individuals with grass pollen allergy at the anticipated peak of the grass pollen season in June/July 2025 (Figure S1). Secondary endpoints included documenting the mean distribution of allergic symptoms using a visual analogue scale (VAS). Nasal and ocular symptoms were also evaluated using VAS, as well as any asthma-related symptoms and treatments. These patient-related outcomes were recorded within an electronic Diary (eDiary), the mobile application CCC Study Diary [3].

In light of the proven side effects of systemic steroids [4], particularly with long-term use, which is necessary for persistent allergic rhinitis—a condition that includes grass pollen allergy—the use of this class of drugs for the indication of allergic rhinitis no longer appears justified. This also calls into question the classic “weighted” CSMS [2], which designated these treatment forms as the third and highest level of therapy. A modified score, hereinafter referred to as the “additive” CSMS, overcomes the disadvantages of the classic medication scale and offers the possibility of using oral antihistamines, nasal glucocorticoids and ocular antiallergics in a targeted, stepwise manner, depending on nasal, ocular or systemic symptoms. Thus, a comparison of both scales was carried out within the framework of this epidemiological study.

This epidemiological study therefore investigated how the severity of the disease is distributed among representative individuals interested in grass pollen AIT.

Consequently, this epidemiological study aimed to investigate the distribution of disease severity among a representative population of individuals expressing interest in grass pollen AIT.

## 2. Materials and Methods

### 2.1. Study Design and Endpoints

This study was a multicentre, prospective, non-interventional epidemiological investigation conducted in Germany. The primary endpoint was the CSMS, averaged over the 30 days of June/July 2025. Secondary endpoints included the daily symptom score (dSS) and daily medication score (dMS), as well as symptom assessments for the eyes, nose, and general allergic manifestations recorded on a VAS.

Asthma symptoms and corresponding medication use were documented using both an asthma scoring system and a VAS.

### 2.2. Setting and Subjects

This epidemiological study was conducted in accordance with §4 paragraph 23 of the German Medicinal Law (AMG) and was funded by Inmunotek S.L. It was performed as a multicentre trial in Germany at outpatient sites specialised in allergology, observing patients with allergic symptoms to grass pollen.

Eligible patients were adults (≥18 years) experiencing moderate to severe allergic rhinitis or rhinoconjunctivitis, with or without controlled asthma, resulting from natural sensitisation to grass pollen. Skin prick tests (SPTs) were performed for each patient following the practical guide for SPTs for aeroallergens [5], with results documented by the investigators.

The study was initially planned for approximately 30 study centres across Germany and was conducted from May 2025 to August 2025 (Table 1). Patients presenting at the study centres in May 2025 were offered participation and provided with detailed information regarding the study procedures by the local medical study director.

Patients who consented to the use of their data underwent a thorough allergy-related medical history during the screening visit. If the patient’s symptoms aligned with guideline-defined profiles, an allergy test was performed, including skin prick tests with grass allergens, house dust mites, a negative control, and histamine as a positive control. Patients sensitised to grasses, with absent or low (clinically non-significant) sensitisation to house dust mites, were included in the study.

Enrolment was completed by providing access credentials for the CCC STUDY Diary app, which was installed on the patient’s mobile device (smartphone or tablet). Patients were instructed to record daily their allergic symptoms (including asthma symptoms, if applicable) and anti-allergic/asthma medication intake in the app from 1 to 30 June 2025. Telephone reminders were made at the beginning of June (telephone contact 1). In August 2025, or once individual average values were available, patients were informed of their recorded results via telephone (telephone contact 2), followed by a detailed discussion to assess whether symptom severity was sufficient to warrant AIT.

To ensure sample representativeness, 22 active study centres aimed to enrol 600 patients, which was considered sufficient to determine statistical distribution measures in the population.

**Inclusion criteria:**

- Male, female, or diverse individuals interested in allergen-specific immunotherapy, with a history of at least 2 previous seasons in which symptomatic therapy was required confirmed by medical history and a positive SPT for grass allergens.
- Age 18–64 years with access to a smartphone or tablet computer.
- Provision of a signed and dated informed consent form for data use.

**Exclusion criteria:**

- Clinically relevant house dust mite allergy confirmed by SPT.
- Lack of access to a smartphone or tablet.

Prior to enrolment, patients were fully informed about the study objectives and signed the informed consent document to confirm agreement for the use of their pseudonymised health data. Patients retained the right to withdraw from the study at any time without providing a reason, with all previously collected data subsequently deleted.

### 2.3. Clinical Endpoints

**Combined Symptom and Medication Score (CSMS)**

The primary endpoint was the statistical distribution of the CSMS in a representative sample of grass pollen allergy sufferers interested in AIT.

The classical weighted CSMS, as defined by Pfaar et al. [2], evaluates the dSS and the dMS equally, with scores ranging from 0 (no symptoms, no need for medication) to 3 (severe symptoms, use of oral corticosteroids). The symptom score includes nasal (rhinorrhoea, sneezing, nasal pruritus, nasal congestion) and ocular (ocular pruritus, watery eyes) symptoms. Based on experience from a previous trial [3], the scoring of dMS in the “additive” CSMS is slightly adapted compared to the scoring method published by Pfaar et al. [2] based on the finding that less than 1% of patients in this study received oral glucocorticosteroids: the use of the following products was permitted in any order or combination and scored additively as follows: antihistamine eye drops (score 0.5), antihistamine tablets (5 mg desloratadine, once daily; score 1.0), nasal corticosteroid (110 µg fluticasone furoate nasal spray per day; score 1.5). Thus, a 7-step scale covering total scores of 0–3 was applied for the determination of the additive dMS. Both dSS and additive dMS.

The daily mean additive CSMS was calculated as the sum of the dSS and the additive dMS, equally weighting allergic symptoms and anti-allergic medication use.

**Daily Symptom Score (dSS)**

Symptoms of nasal allergy (rhinorrhoea, sneezing, nasal itching, nasal congestion) and ocular allergy (ocular itching, watery eyes) were recorded daily via a mobile application during the grass pollen season. Each symptom was rated using a 4-point ordinal scale:

- 0 = no symptoms.
- 1 = mild symptoms (present but minimally noticeable; easily tolerable).
- 2 = moderate symptoms (clearly perceptible; bothersome but tolerable).
- 3 = severe symptoms (difficult to tolerate; interferes with daily activities and/or sleep)

The daily symptom score was calculated as the mean of the six symptom ratings: dSS = (SS1 + SS2 + SS3 + SS4 + SS5 + SS6)/6.

**Additive Daily Medication Score (additive dMS)**

Medication use was scored additively based on the type of medication taken each day:

- 0 points: no medication.
- 1 point: non-sedating oral antihistamines.
- 1.5 points: nasal corticosteroids.
- 0.5 points: antihistamine eye drops.

The dMS was the sum of all medication points recorded on that day, with a possible range of 0–3.

**Mean Daily Additive CSMS**

For each participant, the mean daily additive CSMS was calculated for the period from 1 to 30 June 2025. Descriptive statistics were used to summarise the data, including mean, median, standard deviation, 25th and 75th percentiles, and minimum and maximum values.

**Visual Analogue Scale (VAS)**

Patients rated the severity of their overall allergic symptoms, nasal symptoms, ocular symptoms, and asthma symptoms using a VAS ranging from 0 (not bothersome) to 100 (extremely bothersome). The position marked by the patient was recorded as a numerical value on this scale.

**Asthma Score**

Asthma symptoms, such as breathlessness, coughing, wheezing, and chest tightness, were documented daily using the same 4-point ordinal scale as for nasal and ocular symptoms. The daily asthma symptom score was calculated as the mean of the four symptom ratings:

Asthma score = (SS1 + SS2 + SS3 + SS4)/4.

Asthma scores were analysed only for participants with asthma. The use of asthma inhalation therapy was recorded for all participants as a binary variable (“Yes” or “No”).

### 2.4. Data Sources and Management

The contract research organisation (CRO) was responsible for processing the pseudonymised study data for scientific purposes. Throughout the study and during evaluation of the results, data were processed by the CRO and securely stored on servers hosted by the CRO in Germany.

Electronic data capture (EDC) systems utilised during the study included the CCC STUDY Diary, version 0.101.502 and SPSS Statistics for Windows, version 29.0.1 and 30.0.0.0. (Armonk, NY: IBM Corp.) software for data collection and analysis.

**Observation form:**

One-page observation sheet for documenting the eligibility of patients.

**CCC STUDY Diary App:**

The electronic diary “CCC STUDY Diary” is an application (app) for iOS and Android mobile operating systems. The app was provided to participants free of charge as part of the study. Daily symptoms and medication intake during the grass pollen season were evaluated using the CCC STUDY Diary app.

**List/description of recorded data:**

The following data were collected and documented:

- **Baseline patient characteristics:** Year of birth, age, gender, history of grass pollen allergy symptoms over at least the previous two years, and anti-allergic medication use over the same period.
- **Diagnostic data:** Skin prick test results recorded on the observation form during the screening visit.
- **Daily monitoring data:** CSMS, VAS ratings for general allergy symptoms, nasal and ocular complaints, and asthma symptoms; daily asthma symptom scores; and medication intake, all recorded via the CCC STUDY Diary app.

### 2.5. Statistical Methods

All statistical analyses were conducted using IBM SPSS Statistics for Windows (versions 29.0.1 and 30.0.0.0; Armonk, NY, USA: IBM Corp.) or other validated statistical software. Study endpoints were analysed using descriptive and exploratory statistics.

Continuous variables were summarised using standard statistical measures, including mean, standard deviation, median, minimum, and maximum values. Categorical variables were described using absolute frequencies and percentages of valid cases. Confidence intervals were calculated using the Clopper–Pearson method.

For exploratory comparisons, Student’s *t*-test or Mann–Whitney U test was applied to continuous variables, and Chi-square test or Fisher’s exact test was used for categorical variables. All statistical tests were two-sided, with a significance threshold of *p* = 0.05.

No bias-reduction measures were applied in this observational study. There were no restrictions on the number of patients enrolled per study site; participants were recruited consecutively as they presented for routine care of their grass pollen allergy.

### 2.6. Ethical Supervision

The study was registered in the German Clinical Trials Register (DRKS) under the identifier DRKS00037045 in June 2025. Ethical approval was obtained from the Ethics Committee of the Medical Faculty of the University Hospital Cologne, Cologne, with a positive vote granted on 30 April 2025 (Reference no.: 25-2097).

## 3. Results

**Figure 1:**
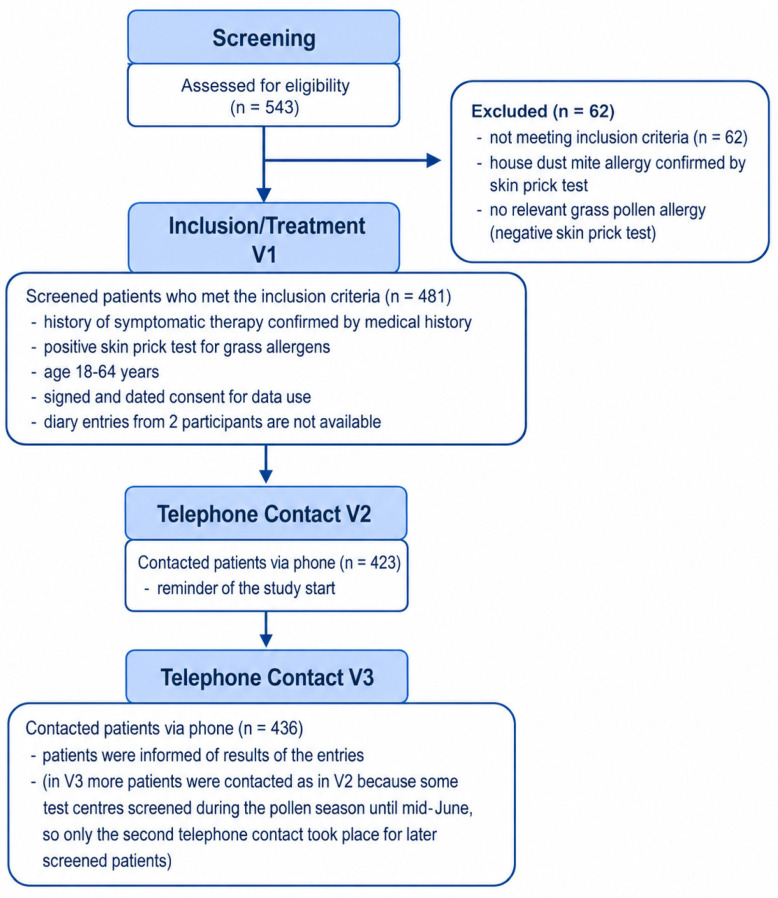
Flow chart of the study.

A total of 600 patients were initially planned for recruitment. Overall, 543 patients were screened, and 481 patients met the eligibility criteria and were enrolled in the study. Sixty-two patients failed the screening and were not included. Two patients did not provide valid eDiary data, and 45 patients missed the second telephone contact (Figure 1).

### 3.1. Baseline Data

**Figure 2:**
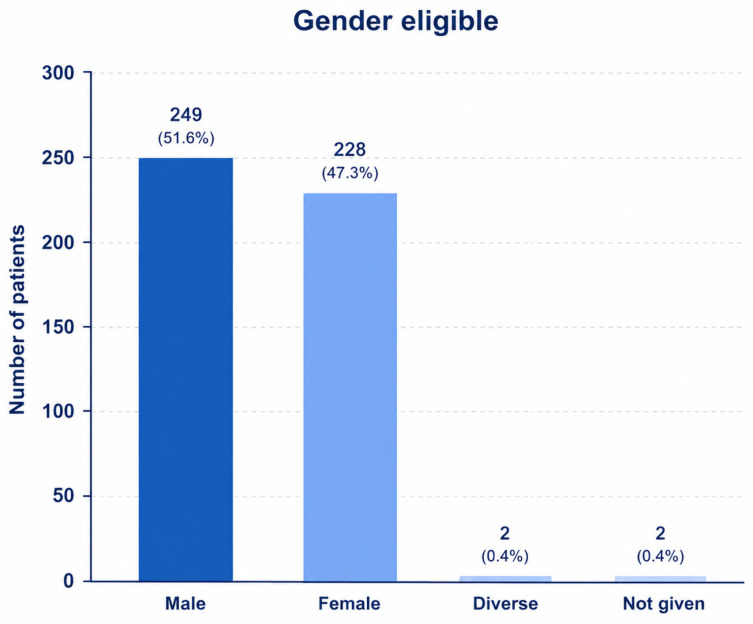
Gender distribution.

The gender distribution among eligible participants was relatively balanced, indicating that the study population can be considered gender-neutral (Figure 2).

**Figure 3:**
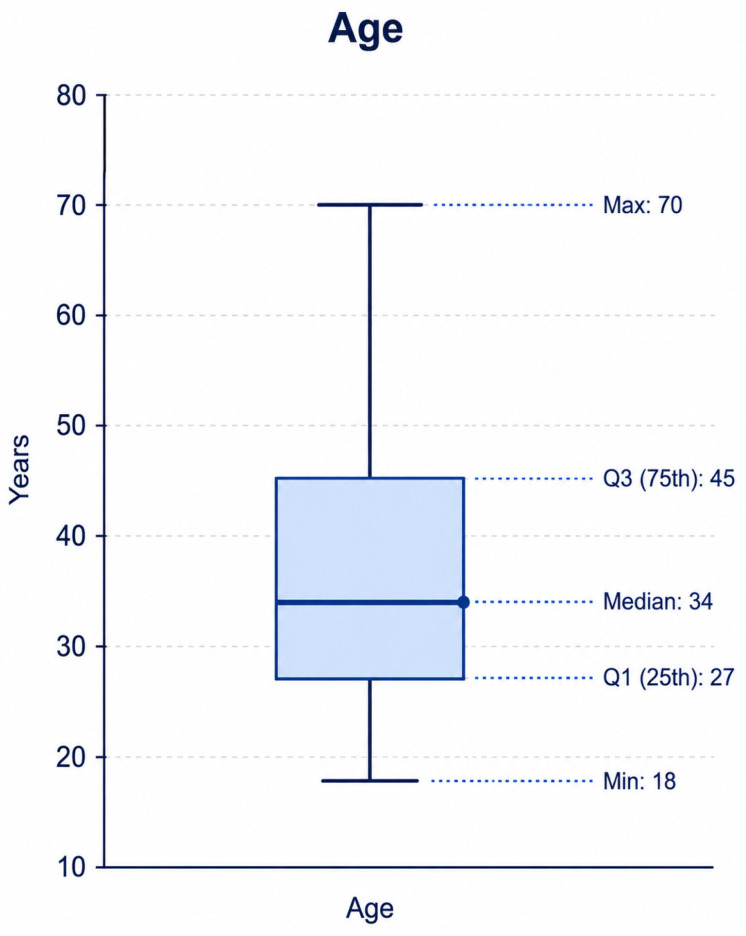
Age distribution. Data is expressed as median and interquartile range (IQR) as well as min/max values.

The age of eligible participants ranged from 18 to 70 years, with a mean age of 36.36 years and a median age of 34 years, resulting in an overall age span of 52 years (Figure 3).

The SPT results of eligible patients are given in Table S1. Two patients were included based on grass pollen-specific IgE (CAP class 3, ≥3.5 kU/L).

### 3.2. Outcome Data

Table 2 presents descriptive statistics for the dSS, additive dMS and additive CSMS based on data from 479 patients with valid diary entries. Both dSS and additive dMS exhibited relatively low variability, whereas the CSMS showed a higher mean and a broader distribution. Percentile and median values indicate a slight skew towards higher scores. The 95% confidence intervals suggest precise estimation of the mean values.

#### 3.2.1. Additive CSMS

**Figure 4:**
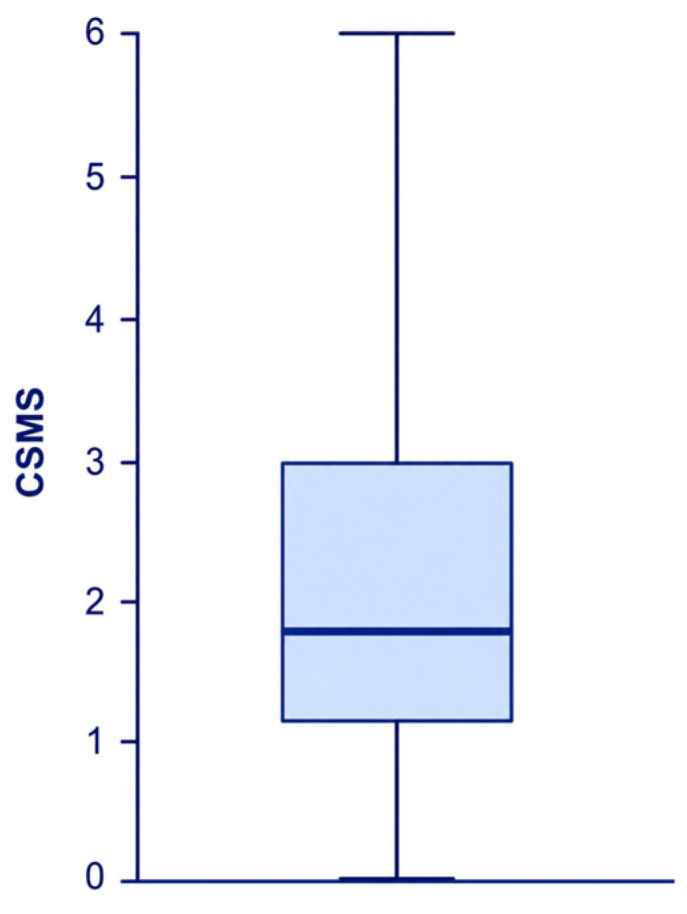
Boxplot showing the CSMS. Data is expressed as the median CSMS + IQR, as well as min/max values.

The boxplot of the additive CSMS (Figure 4) demonstrates a broader distribution compared with the individual plots for dSS and additive dMS. The median additive CSMS was 1.88, with a lower quartile (P25) of 1.13 and an upper quartile (P75) of 2.99. Values ranged from 0.02 to 6.00, with a notably extended upper whisker, reflecting the heterogeneity of the study population. The entire interquartile range lies above 1.0, indicating that the majority of patients experienced moderate to relatively high overall symptom and medication scores.

**Figure 5:**
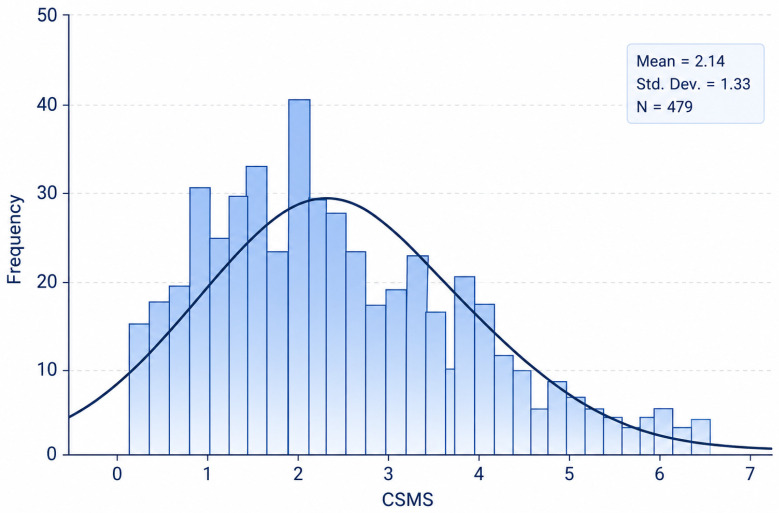
Distribution curve of the CSMS in steps of 0.2.

The distribution curve of the CSMS exhibits a right-skewed pattern with a wide range of values, with a mean of 2.14. This confirms the heterogeneous distribution previously indicated by the boxplot (Figure 4 and Figure 5).

The suitability of patients for AIT was evaluated using the CSMS, with a predefined threshold of ≥1.5 set as the criterion for treatment eligibility. In the present study, the majority of participants, 63.3% (n = 303), met the CSMS threshold of ≥1.5.

#### 3.2.2. dMS

**Figure 6:**
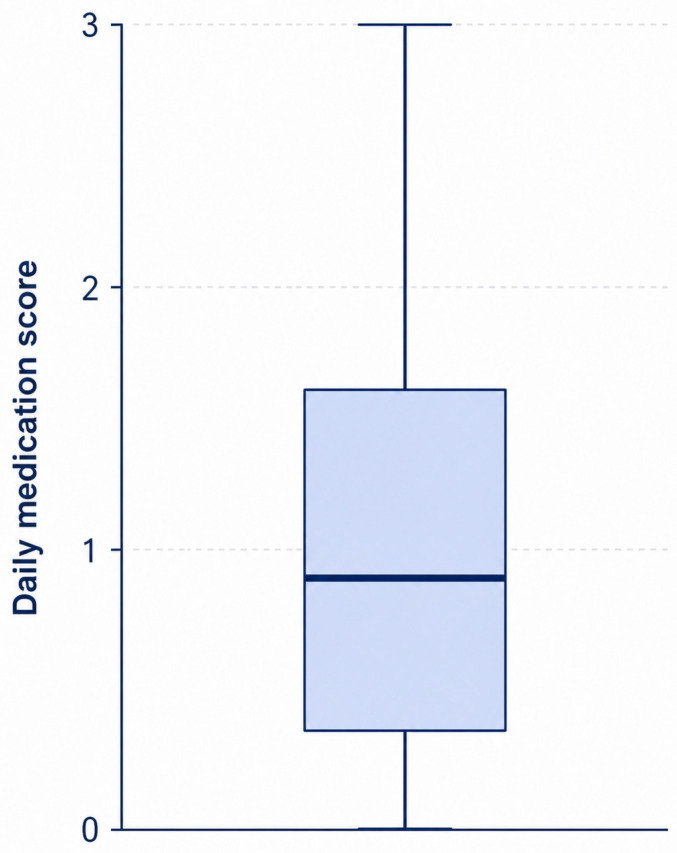
Boxplot showing the dMS. Data is expressed as the median CSMS + IQR, as well as min/max values.

The boxplot for the dMS reveals a median of 0.93, with the lower quartile (P25) at 0.30 and the upper quartile (P75) at 1.66. Values ranged from 0.00 to 3.00. Compared with the dSS boxplot, the dMS box shows a greater vertical spread, reflecting higher variability in medication use (Figure 6).

**Figure 7:**
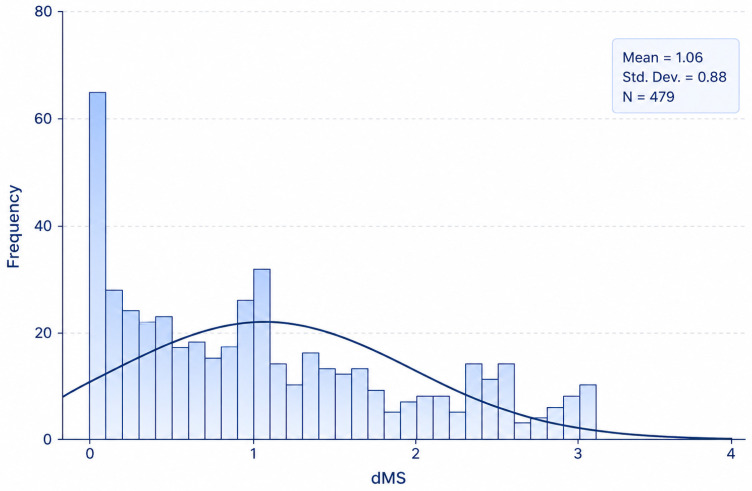
Distribution curve of dMS in steps of 0.1.

The distribution curve of the dMS demonstrates a clear clustering of values below a dMS of 1.5. The gradual decrease in frequency up to the maximum value of 3.00 corresponds with the moderate variability observed in the boxplot (Figure 7).

#### 3.2.3. dSS

**Figure 8:**
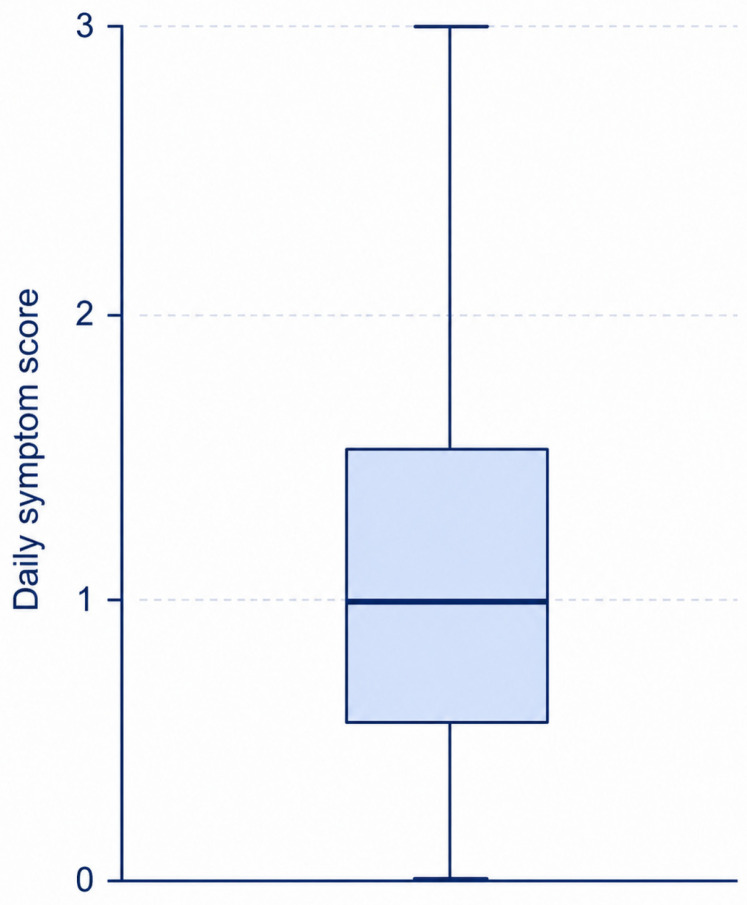
Boxplot showing the dSS. Data is expressed as the median CSMS + IQR, as well as min/max values.

The boxplot of the dSS shows a largely homogeneous distribution. The median is 0.94 with lower quartile (P25) of 0.59 and upper quartile (P75) of 1.49. The values range from a minimum of 0.02 to a maximum of 3.00. The size of the box is the most compact of the three listed plots (CSMS, dMS, dSS), indicating a moderate distribution of symptom severity (Figure 8).

**Figure 9:**
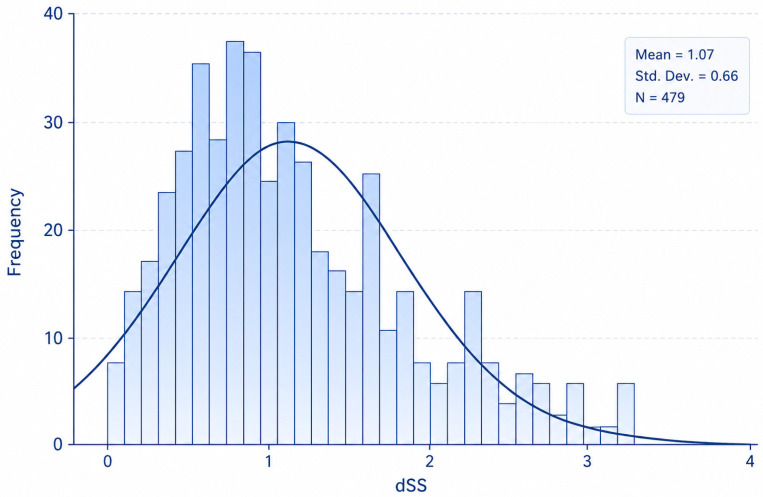
Distribution curve of dSS in steps of 0.1.

The distribution of the dSS corroborates the lower variability indicated by the boxplot. Values cluster around the median of 0.94, with scores above 2.00 occurring relatively infrequently. The curve exhibits a short, right-skewed tail, with a mean of 1.07 (Figure 9).

### 3.3. Comparison of the Two Methods of CSMS Collection (Additive vs. Classical)

To further validate the reliability of the CSMS calculation, a sensitivity analysis was conducted, comparing the classical weighted and additive methods for the CSMS and dMS [3,5].

*Weighted Medication score* [2].

1 point—Oral and/or topical (eyes or nose) nonsedative H1 antihistamines H1A.
2 points—Intranasal corticosteroids (INS) with/without H1A.
3 points—Oral corticosteroids with/without INS, with/without H1A.
(Total) Daily medication score (dMS) 0–3 (max score is 3).

Both calculation methods were applied in parallel for all 479 evaluated patients. Descriptive statistics demonstrate similar characteristics across methods: the mean additive CSMS was 2.14, while the weighted CSMS had a mean of 1.98. For the daily medication score, the mean additive dMS was 1.06 compared with 0.91 for the weighted dMS (Table 3).

The Wilcoxon tests for both analyses yielded *p*-values of <0.001, indicating statistically significant differences; however, these differences were not considered clinically relevant. Correlations between the weighted and additive scores were very strong, with Spearman’s rho of 0.983 for the dMS and 0.990 for the CSMS (*p* < 0.001). Linear regression analyses further confirmed an almost perfect correlation, with β-values of 0.966 for the dMS and 0.986 for the CSMS (Table 4).

**Figure 10:**
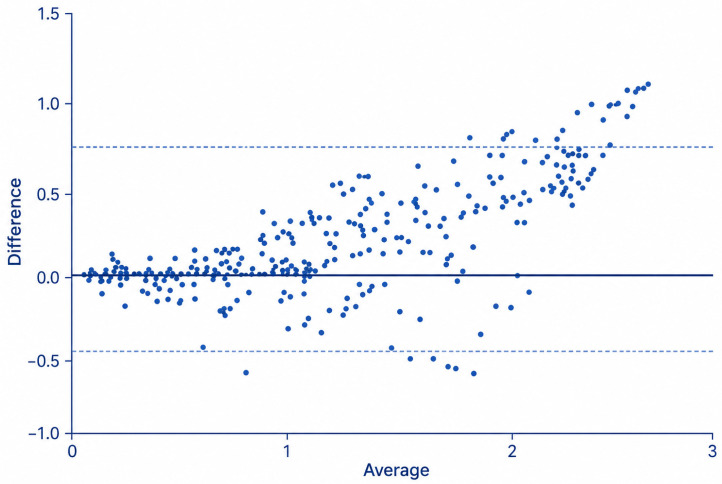
Bland–Altman plot for comparing weighted and additive CSMS values.

The Bland–Altman analysis further confirmed strong methodological agreement, with a low bias of 0.157 (SD: 0.2970) and 95% limits of agreement ranging from −0.425 to 0.739. Notably, 95% of the data points fell within the accepted limits, demonstrating a high degree of consistency between the two calculation approaches (Figure 10).

**Mean Difference (Bias):** A value close to zero indicates good agreement. In our analysis, the bias was low, at 0.157.

**Limits of Agreement (LoA):** The dashed lines represent the upper and lower limits of agreement (mean ± 1.96 × SD). If the methods are in good agreement, most data points should fall within these limits. In this study, the limits of agreement differences were small, with a total of 1.16 and a mean of 0.58, indicating only minor discrepancies between the methods.

**Spread:** The vertical dispersion of points illustrates the variability in differences. A consistent spread across the range of means indicates homoscedasticity (constant variance). In this study, the variability increases with higher measurements. Notably, there were no values above 5 in the weighted group, whereas the additive method reached a maximum value of 6.

**Outliers:** Points falling outside the limits of agreement may indicate disagreement or measurement issues. In this dataset, only a few outliers were observed, fewer than 5%, primarily occurring at higher values.

**Figure 11:**
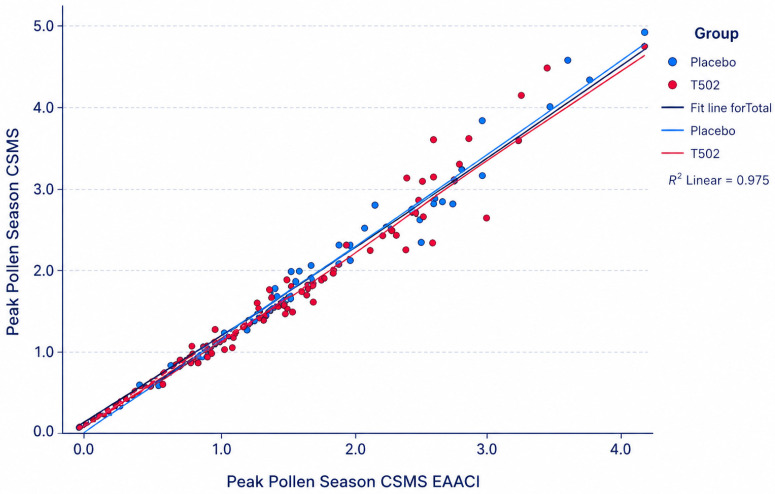
Correlation of weighted and additive CSMS values (previous analysis of the T502-SIT-045 trial [6]).

These results confirm that the CSMS provides a valid basis for assessing both symptom severity and potential AIT treatment (Figure 11).

### 3.4. Other Analyses

#### 3.4.1. Severity of Allergy Symptoms (Recorded by Visual Analogue Scale, VAS)

In addition to the CSMS, the severity of allergic symptoms was assessed using a VAS, ranging from 0 (no symptoms) to 100 (unbearable symptoms) (Table S2 in the Supplementary Material).

#### 3.4.2. Asthma

Of the 479 patients, 172 reported asthma as a comorbid condition (Table S3 in the Supplementary Material). The use of asthma medication was documented for all patients.

## 4. Discussion

The link between allergic symptoms and exposure to grass pollen is well established.

Various studies showed a direct link between the concentration of pollen in the air (pollen count) and the severity of symptoms (nasal, ocular, bronchial) in affected individuals.

Landesberger et al. [7] demonstrated a statistically significant correlation between grass pollen concentration and the severity of physical symptoms (eyes/nose) as well as impairment in daily life. Luyten et al. [8] investigated the non-linear relationship between pollen exposure and symptoms, with a particularly sharp increase in symptom severity at levels above 80 pollen grains/m^3^. Bastl et al. [9] demonstrated the temporal variability of symptoms throughout the grass pollen season and their peaks. In a post hoc analysis of various studies on sublingual AIT for grass pollen allergy, Durham et al. [10] found that the extent of the therapy’s efficacy is linked to the level of pollen exposure. During the assessment period of our study moderate to severe grass pollen exposure occurred as shown in Figure S1.

In a multi-year study of birch pollen allergy sufferers, Novak et al. [11] demonstrated that CSMS values of approximately 2.0 were observed in the placebo group, whilst this outcome parameter was consistently below 1.5 in those treated with depigmented allergoids and was thus significantly lower. The mean values measured in our study therefore correspond to the placebo range. CSMS values in the range between 1.5 and 2.0 are also reported in the conclusive study by Zielen et al. [12], as well as in the successful predecessor study by de Kam et al. [13] published two years earlier.

A total of 600 patients were planned for inclusion in the study. Overall 543 individuals were screened, and 481 ultimately met all inclusion criteria, with 479 providing valid diary data for the final analysis.

SPT for grass pollen and house dust mite was performed in 541 screened patients. Results demonstrated that grass pollen allergens induced the largest mean wheal diameter of 8.06 mm, exceeding the positive control by 1.9 mm, thereby confirming the predominance of grass pollen sensitisation in this cohort. This is in line with a moderate to severe grass pollen sensitisation according to Malling et al. [14].

Comparison of the dSS and additive dMS revealed an interesting behavioural pattern. A subgroup of patients consistently experienced moderate to severe symptoms during the pollen season but reported lower or inconsistent use of medication. This may reflect suboptimal adherence or a limited perceived need for pharmacotherapy, potentially resulting in a skewed representation of medically optimised symptom control. This is in contrast to Murphy et al. [15] who observed a decoupling of dSS but not of dMS from grass pollen exposure.

Analysis of the additive CSMS yielded a mean of 2.14 and a median of 1.88, indicating moderate symptom severity for the majority of participants. Overall, 63.3% (n = 303) of patients met the predefined CSMS threshold of ≥1.5, confirming their at least moderate disease severity and by consequence [15] their suitability for AIT. Conversely, this means that more than one in three patients who are interested in AIT and are deemed suitable by an experienced allergist would probably be better off with symptomatic treatment [16].

Sensitivity analyses were conducted to assess the robustness of CSMS calculations, comparing the weighted method (according to EAACI [2]) with the additive approach [6] for both the CSMS and dMS. High methodological consistency was observed, with Spearman’s rho values of 0.983 for the dMS and 0.990 for the CSMS (both *p* < 0.001). Wilcoxon tests indicated statistically significant, though not clinically relevant, differences. Bland–Altman analysis further confirmed a strong correlation, with a low bias of 0.157 and 95% limits of agreement from −0.425 to 0.739. Similar, albeit slightly modified, approaches were investigated as part of the MASK-air initiative, with a strong correlation found with the VASs used [17,18,19,20].

Severity of allergic symptoms was additionally assessed using a VAS, which produced a mean total score of 41.81, reflecting moderate overall symptom intensity under symptomatic anti-allergic treatment [17]. Asthma was reported as a comorbidity in 172 of 479 patients (36%), generally associated with mild to moderate daily respiratory symptoms.

### 4.1. Limitations

This study has several limitations related to the assessment of subjective experiences. The self-reported nature of the data may introduce recall and response biases, as participants’ perceptions can vary over time and may be influenced by social desirability, potentially leading to overestimation of symptoms. Contextual factors such as mood, environment, or the timing of data entry may also have affected participants’ ratings.

Allergic rhinitis is a condition that significantly impairs patients’ quality of life, as documented in numerous publications [1,2,3,6,12,17]. In our study, quality of life was not assessed due to the scope limitations of the epidemiological design. However, it has been shown that both symptomatic treatments, as applied in our study and AIT subsequently recommended to patients after the study period can lead to a substantial improvement in patients’ perceived quality of life [21].

Another limitation is the occurrence of dropouts, which could introduce attrition bias. However, only two patients lacked valid diary data, representing a minimal proportion that is unlikely to affect the representativeness or generalisability of the results. Medication adherence could not be independently verified, meaning that insufficient compliance may have contributed to persistent symptoms. Consequently, self-reported entries may overstate symptom severity compared with outcomes observed under optimised therapy, though this remains speculative.

Additionally, the study did not achieve the initially planned number of medical centres, enrolling 22 instead of the target 30, which may have limited regional diversity. Similarly, the final sample of patients with valid diary data was 479 rather than the planned 600, slightly reducing the statistical power of the study.

### 4.2. Interpretation

The comparative evaluation of additive and weighted CSMS and dMS calculations demonstrated that, although minor statistical differences were detectable, both scoring approaches yield nearly identical clinical interpretations. The correlation between the two methods was very high, and the Bland–Altman analysis confirmed good agreement, indicating strong consistency in assessing symptom severity.

From a clinical perspective, these findings support the reliability of the CSMS as an indicator of AIT eligibility. Considering the descriptive statistics, with a CSMS median of 1.88 and a lower quartile (P25) of 1.13, a substantial proportion of the evaluated individuals experience moderate to severe symptoms of grass pollen allergy, based on the predefined threshold of ≥1.5. The methodological consistency between the additive and weighted calculation approaches strengthens confidence in the representativeness, robustness, and validity of the study results, thereby supporting the use of CSMS as a practical, real-world tool for patient evaluation in allergy diagnostics and treatment decision making.

The findings further highlight that symptom intensity and corresponding medication use are closely aligned, reinforcing the utility of CSMS for identifying candidates likely to benefit from AIT in routine clinical practice. Moreover, the strong agreement between the additive and weighted approaches indicates that the study’s conclusions regarding AIT eligibility are robust, irrespective of the specific calculation method employed.

### 4.3. Generalisability

The study was conducted across 22 allergology-specialised centres in Germany. The demographic characteristics, including the age range and balanced gender distribution, support moderate generalisability of the findings to adult patients with grass pollen allergy in Germany, particularly to those seeking care in specialised clinical centres.

## 5. Conclusions

This study successfully characterised the symptom burden and treatment eligibility of patients with grass pollen allergy. Analysis of 479 patients confirmed that the CSMS appears as a valid and reproducible measure of disease severity. A CSMS threshold of ≥1.5 may help to identify patients with moderate to severe symptoms suitable for AIT. Sensitivity analyses demonstrated strong agreement between weighted and additive CSMS and daily medication scores. Despite minor limitations, these findings provide robust real-world evidence supporting the CSMS as a reliable tool for assessing AIT eligibility.

## Figures and Tables

**Table 1:** Timetable for individual study participants.

Timepoint	Visit	Assessments
**May 2025**	Screening visit	Introduction at the study centre with information, consent, allergen-related medical history and previous anti-allergic medication use, skin prick test and, if suitable, activation of the CCC STUDY Diary app.
**June 2025**	Telephone contact 1	Beginning of June to start entries and documentation in the app from 1 June 2025 to 30 June 2025.
**August 2025**	Telephone contact	Telephone consultation, discussion of the results from the app.

**Table 2:** Descriptive statistics of dSS, additive dMS, and additive CSMS.

	dSS	dMS	CSMS
Count	479	479	479
Valid N	479	479	479
Mean	1.07	1.06	2.14
Standard Deviation	0.66	0.88	1.33
Maximum	3.00	3.00	6.00
Median	0.94	0.93	1.88
Minimum	0.02	0.00	0.02
Percentile 25	0.59	0.30	1.13
Percentile 75	1.49	1.66	2.99
95.0% Lower CL for Mean	1.02	0.99	2.02
95.0% Upper CL for Mean	1.13	1.14	2.26

**Table 3:** Descriptive statistics of weighted and additive CSMS and dMS.

	dMS		CSMS	
	Additive	Weighted	Additive	Weighted
Total N	479	479	479	479
Valid N	479	479	479	479
Mean	1.06	0.91	2.14	1.98
Standard Deviation	0.88	0.66	1.33	1.12
Standard Error of Mean	0.04	0.03	0.06	0.05
Minimum	0.00	0.00	0.02	0.02
Percentile 25	0.30	0.32	1.13	1.12
Median	0.93	0.88	1.88	1.86
Percentile 75	1.66	1.47	2.99	2.73
Maximum	3.00	2.00	6.00	5.00
*p* *	<0.001		<0.001	
Spearmans rho	0.983		0.990	
*p* **	<0.001		<0.001	

* Wilcoxon test, ** Spearman correlation.

**Table 4:** Linear regression analysis of dMS and CSMS.

Coefficients					
Model	Unstandardised Coefficients		Standardised Coefficients	t	Sig.
	B	Std. Error	Beta		
(Constant)	−0.109	0.018		−6.169	<0.001
dMS_weighted_mean	1.293	0.016	0.966	82.079	<0.001
a. Dependent Variable: dMS_additive_mean					
(Constant)	−0.186	0.021		−8.917	<0.001
CSMS_weighted_mean	1.173	0.009	0.986	127.924	<0.001
a. Dependent Variable: CSMS_additive_mean					

## Data Availability

Data will be available from the corresponding author upon reasonable request.

## Supplementary Materials

The following supporting information can be downloaded at https://www.mdpi.com/article/10.3390/jcm15114035/s1: Figure S1. Daily Grass Pollen forecast for Germany as published by Deutscher Wetterdienst (DWD). Table S1. Descriptive Statistics of SPT results in mm (eligible patients), Table S2: Descriptive Statistics of the VAS Severity of allergy symptoms, Table S3: Descriptive Statistics of Asthma as an additional disease.

## Abbreviations

The following abbreviations are used in this manuscript:

AIT	Allergen Immunotherapy
AMG	German Medicines Act (Arzneimittelgesetz)
CCC	ClinCompetence Cologne GmbH
CRO	Clinical Research Organisation
CSMS	Combined Symptom–Medication Score
dSS	Daily Symptom Score
dMS	Daily Medication Score
DRKS	German Clinical Trials Register
EAACI	European Association of Allergy and Clinical Immunology
eCRF	Electronic Case Report Form
eDiary	Electronic Patient Diary
EDC system	Electronic Data Capture System
IgE	Immunoglobulin E
IQR	Interquartile Range
NIS	Non-Interventional Study
*p*	(*p*-value) Value for Significance
P25	Percentile 25
P75	Percentile 75
SPT	Skin Prick Test
VAS	Visual Analogue Scale

## References

[1] Bachert C., Hoogeveen H., Have R.T., Yu D., Worm M., Pfaar O., Jutel M., Distler A., Bozek A., Opstelten D. (2025). Subcutaneous Allergen Immunotherapy in Adults Allergic to House Dust Mites: A Phase 3 Randomized Controlled Trial. Allergy.

[2] Pfaar O., Demoly P., van Wijk R.G., Bonini S., Bousquet J., Canonica G.W., Durham S.R., Jacobsen L., Malling H.J., Mösges R. (2014). Recommendations for the standardization of clinical outcomes used in allergen immunotherapy trials for allergic rhinoconjunctivitis: An EAACI Position Paper. Allergy.

[3] Mösges R., Zeyen C., Raskopf E., Acikel C., Sahin H., Allekotte S., Cuevas M., Shamji M.H., Subiza J.L., Casanovas M. (2023). A randomized, double-blind, placebo-controlled trial with mannan-conjugated birch pollen allergoids. Allergy.

[4] Hox V., Lourijsen E., Jordens A., Aasbjerg K., Agache I., Alobid I., Bachert C., Boussery K., Campo P., Fokkens W. (2020). Benefits and harm of systemic steroids for short- and long-term use in rhinitis and rhinosinusitis: An EAACI position paper. Clin. Transl. Allergy.

[5] Bousquet J., Heinzerling L., Bachert C., Papadopoulos N.G., Bousquet P.J., Burney P.G., Canonica G.W., Carlsen K.H., Cox L., Haahtela T. (2012). Practical guide to skin prick tests in allergy to aeroallergens. Allergy.

[6] Mösges R., Raskopf E., Klimek L., Pfaar O., Zielen S., Xenofontos E., Decker L., Neuhof C., Rybachuk A., Acikel C. (2025). Short-course subcutaneous treatment with birch pollen allergoids greatly improves symptom and medication scores in birch allergy. Allergy.

[7] Landesberger V., Huß J., Grenzebach K., Nowak D., Gröger M., Oppel E., Schaub B., French L.E., Kutzora S., Quartucci C. (2025). Association of grass pollen concentration and physical symptoms as well as impairments in day-to-day life in pollen allergy patients. Sci. Rep..

[8] Luyten A., Bürgler A., Glick S., Kwiatkowski M., Gehrig R., Beigi M., Hartmann K., Eeftens M. (2024). Ambient pollen exposure and pollen allergy symptom severity in the EPOCHAL study. Allergy.

[9] Bastl M., Bastl K., Dirr L., Berger M., Berger U. (2021). Variability of grass pollen allergy symptoms throughout the season: Comparing symptom data profiles from the Patient’s Hayfever Diary from 2014 to 2016 in Vienna (Austria). World Allergy Organ. J..

[10] Durham S.R., Nelson H.S., Nolte H., Bernstein D.I., Creticos P.S., Li Z., Andersen J.S. (2014). The magnitude of efficacy measurements in grass allergy immunotherapy trials is highly dependent on pollen exposure. Allergy.

[11] Novak N., Worm M., Staubach P., Jutel M., Sager A., Pfaar O. (2022). Subcutaneous birch pollen allergen immunotherapy with a depigmented polymerised extract shows only sustained and long-term efficacy in a subgroup of monosensitised adults and adolescents with allergic rhinitis. Clin. Transl. Allergy.

[12] Zielen S., Bernstein J.A., Sturm G.J., Jutel M., Pfaar O., Shamji M.H., Mösges R., Berger M., Berger U.E., RESONATE Investigator Group (2025). Six Injections of Modified Adjuvanted PQ Grass Is Effective and Well-Tolerated in a Pivotal Phase III Trial. Allergy.

[13] de Kam P.J., Zielen S., Bernstein J.A., Berger U., Berger M., Cuevas M., Cypcar D., Fuhr-Horst A., Greisner W.A., Jandl M. (2023). Short-course subcutaneous treatment with PQ Grass strongly improves symptom and medication scores in grass allergy. Allergy.

[14] Malling H., Montagut A., Melac M., Patriarca G., Panzner P., Seberova E., Didier A. (2009). Efficacy and safety of 5-grass pollen sublingual immunotherapy tablets in patients with different clinical profiles of allergic rhinoconjunctivitis. Clin. Exp. Allergy.

[15] Murphy K., Gawchik S., Bernstein D., Andersen J., Pedersen M.R. (2013). A phase 3 trial assessing the efficacy and safety of grass allergy immunotherapy tablet in subjects with grass pollen-induced allergic rhinitis with or without conjunctivitis, with or without asthma. J. Negat. Results Biomed..

[16] Datz N., Arens A., Kordonouri D.M.O. (2015). Die Hyposensibilisierung bleibt nur selten als Therapieoption übrig. Hautnah Dermatol..

[17] Bousquet J., Sousa-Pinto B., Anto J.M., Bedbrook A., Czarlewski W., Ansotegui I.J., Bergmann K., Braido F., Brussino L., Cecchi L. (2024). Concurrent validity, cut-offs and ability to change of patient-reported outcome measures for rhinitis and asthma in MASK-air^®^. Clin. Transl. Allergy.

[18] Sousa-Pinto B., Azevedo L.F., Jutel M., Agache I., Canonica G.W., Czarlewski W., Papadopoulos N.G., Bergmann K., Devillier P., Laune D. (2022). Development and validation of combined symptom-medication scores for allergic rhinitis. Allergy.

[19] Kvedarienė V., Biliute G., Didziokaitė G., Kavaliukaite L., Savonyte A., Rudzikaite-Fergize G., Puronaite R., Norkuniene J., Emuzyte R., Dubakiene R. (2022). Mobile health app for monitoring allergic rhinitis and asthma in real life in Lithuanian MASK-air users. Clin. Transl. Allergy.

[20] Sousa-Pinto B., Schünemann H.J., Sá-Sousa A., Vieira R.J., Amaral R., Anto J.M., Klimek L., Czarlewski W., Mullol J., Pfaar O. (2022). Consistent trajectories of rhinitis control and treatment in 16,177 weeks: The MASK-air^®^ longitudinal study. Allergy.

[21] Olivier C.E., dos Santos Lima R.P., Argentão D.G.P., da Silva M.D., dos Santos R.A.P.G., Pensuti M., Piai-de-Morais T.H. (2013). Group-specific Multi-allergen Sublingual/Swallow Immunotherapy Improves the Quality of Life of Polysensitized Children and Adults with Allergic Rhinitis. J. Allergy Ther..

