# Supplementary material for: Prospective Evaluation of Symptom Burden and Medication Use in Seasonal Allergic Rhinitis/Rhinoconjunctivitis Patients Considering Allergen-Specific Immunotherapy
Source: J Clin Med. 2026 May 22;15(11):4035. doi: 10.3390/jcm15114035 (PMC13258035; doi:10.3390/jcm15114035)
Supplement: Supplementary file 1 [file jcm-15-04035-s001.zip › jcm-4265055-supplementary.pdf]

Journal of Clinical Medicine

Supplementary Materials: The following supporting information can be downloaded at https://www.mdpi.com/article/doi/s1: Figure S1. Daily Grass Pollen forecast for Germany as published by Deutscher Wetterdienst (DWD). Table S1. Descriptive Statistics of SPT results in mm (eligible patients), Table S2: Descriptive Statistics of the VAS Severity of allergy symptoms, Table S3: Descriptive Statistics of Asthma as an additional disease.

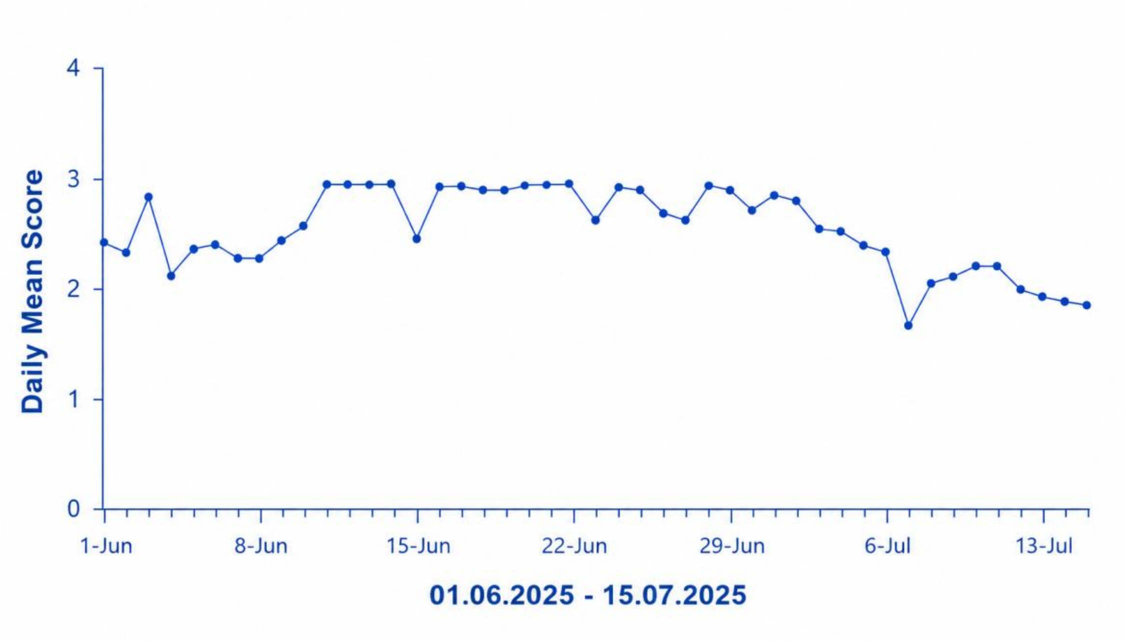

Figure S1. Daily Grass Pollen forecast for Germany as published by Deutscher Wetterdienst (DWD).

Specific exposure levels for grass pollen (daily mean concentrations, pollen per m³ of air):

Scores:

0: No exposure

1: Up to 5 pollen/m³: Low exposure

2: 6–30 pollen/m³: Moderate exposure

3: More than 30 pollen/m³: High exposure

Table S1. Descriptive Statistics of SPT results in mm (eligible patients).

	Negative control	Positive control	Grass pollen	House dust mite
Valid N	480	479	479	480
Mean	0.04	6.34	8.43	0.92
Standard Deviation	0.27	7.82	8.59	1.66
Minimum	0.00	2.00	3.00	0.00
Median	0.00	5.00	7.00	0.00
Maximum	3.00	90.00	100.00	12.00
Percentile 25	0.00	4.00	5.00	0.00
Percentile 75	0.00	7.00	10.00	2.00

95,0% Lower CL for Mean	0.02	5.63	7.66	0.78
95,0% Upper CL for Mean	0.07	7.04	9.20	1.07

Table S2: Descriptive Statistics of the VAS Severity of allergy symptoms.

	How severe are your allergy symptoms today?	How much do your nasal symptoms affect you today?	How much do your eye symptoms affect you today?
Count	479	479	479
Valid N	479	479	479
Mean	41.76	41.58	34.01
Standard Deviation	25.03	25.08	26.93
Maximum	100	100	100
Median	39.43	40.82	27.93
Minimum	0	0.03	0
Percentile 25	21.66	21.5	11.17
Percentile 75	60.27	59.79	53.5

Table S3: Descriptive Statistics of Asthma as an additional disease.

	Asthma - Shortness of breath?	Asthma - Coughing?	Asthma - Wheezing?	Asthma - Tightness in the chest?	How severe are your asthma symptoms today?	Have you used your asthma inhaler today?*
Count	479	479	479	479	479	479
Valid N	172	172	172	172	172	479
Mean	0.79	1.23	0.79	0.83	49.16	4.14
Standard Deviation	0.71	0.75	0.75	0.71	21.23	9.26
Maximum	2.73	3	3	2.8	98.2	30.00
Median	0.75	1.18	0.67	0.81	52.58	0.00
Minimum	0	0	0	0	0	0.00
Percentile 25	0	0.75	0	0.11	33.9	0.00
Percentile 75	1.18	1.76	1.29	1.27	66.5	0.00

*For this question, all participants were included in the analysis independent of asthma.
